# Exploring Fatalities and Injuries in Construction by Considering Thermal Comfort Using Uncertainty and Relative Importance Analysis

**DOI:** 10.3390/ijerph18115573

**Published:** 2021-05-23

**Authors:** Minsu Lee, Jaemin Jeong, Jaewook Jeong, Jaehyun Lee

**Affiliations:** Department of Safety Engineering, Seoul National University of Science and Technology, 232 Gongneung-ro, Nowon-gu, Seoul 01811, Korea; alstn9881@seoultech.ac.kr (M.L.); ss96011@seoultech.ac.kr (J.J.); archi0528@seoultech.ac.kr (J.L.)

**Keywords:** physiological equivalent temperature, fatal accident, outdoor thermal comfort, monte carlo simulation, deep learning

## Abstract

Fatal injury and accidents in the construction industry occur under the influence of outdoor weather conditions such as temperature, humidity and wind speed in all four seasons. Previous research in this area has focused on hot and cold weather conditions: hot weather causes heat rash, heat cramps and heat fainting, while cold weather causes fatigue, lumbago, and cold finger sensations. However, other weather conditions are also associated with, and cause, fatal injury and accidents. Accordingly, this study analyzes injury and fatal accidents in the construction industry based on the physiological equivalent temperature (PET) as it pertains to thermal comfort using an uncertainty analysis. Furthermore, using a neural network, relative importance is analyzed considering injury and fatal accidents. This study is conducted in five steps: (i) Establishment of the database, (ii) Classification of accident types and weather conditions, (iii) Calculation of thermal comfort, (iv) Analysis of injury and fatal accidents based on thermal comfort, and (v) Calculation of the relative importance of thermal comfort during injury and fatal accidents. Via the research process, 5317 fatal incidents and 207,802 injuries are analyzed according to 18 accident types in all seasons. It was found that ‘falls’, were the most frequent fatal incident and injury (2804 fatal incidents and 71,017 injuries), with most of these occurring during the autumn season. The probabilities of injury and fatal accidents in the ‘fall’ category are 86.01% and 85.60%, respectively, in the outside comfort ranges. The contribution of this study can provide data for a database on safety management considering weather conditions.

## 1. Introduction

In contrast to manufacturing industry, the construction industry is such that outdoor work is unrelated to indoor environmental quality (IEQ) measures such as temperature and humidity. Moreover, factors of outdoor environmental quality (OEQ), such as temperature, humidity, wind speed, vibrations, noise, fine dust, and ultrafine dust, significantly affect the performance of construction workers [1,2,3]. For instance, high temperatures can cause construction workers to suffer heat rash, heat cramping, heat fainting, heat exhaustion, and heatstroke [4]. These issues can be related to fatal incidents or injuries caused by physical fatigue, mental disorder, slow response speeds, and poor reasoning abilities [5]. On the other hand, cold temperatures in winter can cause fatigue, lumbago, cold finger sensation, rigidity of the body, and shoulder and neck stiffness [6]. It has been reported that work conditions under 10 °C have a negative influence on concentration, memory, and performance capacity [7]. In this regard, it can be assumed that uncomfortable work conditions are commonly based on weather. These weather conditions are related to injury and fatal accidents. Accordingly, this research focuses on the thermal comfort of construction workers considering their work conditions, such as OEQ. Studies related to OEQ found that injury and fatal accidents occurred due to different temperature conditions and seasonal changes [4,5,6,7,8,9,10,11,12,13,14,15,16,17,18,19,20,21,22,23]. Earlier work analyzed the causes of injury and fatal accidents in relation to specific weather conditions, such as those in the summer and winter seasons [24,25,26,27,28,29,30,31,32,33,34,35,36,37]. Other research analyzed ‘falls’ and ‘slips’ as frequent types of accident which can be affected by weather conditions [36,37,38,39,40,41,42,43,44,45]. Still others analyzed construction productivity using the predicted mean vote (PMV) by surveying the thermal comfort of construction workers [46,47]. Despite these studies, however, the relationship between injury and fatal accidents and thermal comfort is difficult to find in the literature.

Therefore, this study aims to analyze the relationship between injury and fatal accidents and weather conditions in the construction industry using the concept of physiological equivalent temperature (PET) and an uncertainty analysis. In addition, a neural network analysis and relative importance analysis are applied to determine reliable factors that affect the input PET.

## 2. Literature Review

As indicated in Table 1, previous research in this area falls into three categories: (i) research related to the causes of construction disasters [24,25,26,27,28,29,30,31,32,33,34,35,36,37,38,39,40,41,42,43,44,45], (ii) research related to an analysis of the weather impact on injury and fatal accidents in construction [4,5,6,7,8,9,10,11,12,13,14,15,16,17,18,19,20,21,22,23,24,25,26,27,28,29,30,31,32,33,34,35,36,37], and (iii) research related to measuring the thermal comfort of construction workers [46,47,48].

The limitations of previous research are explained below. First, there are various types of accident in construction; however, most previous research has focused on specific accident types, such as ‘disease’, ‘fall’, and ‘drowned’. Second, previous research has focused on specific weather conditions, such as heat wave. Third, although previous studies found that critical weather affected injury and fatal accidents, the important variables of weather impact were not analyzed.

## 3. Materials and Methods

**Figure 1:**
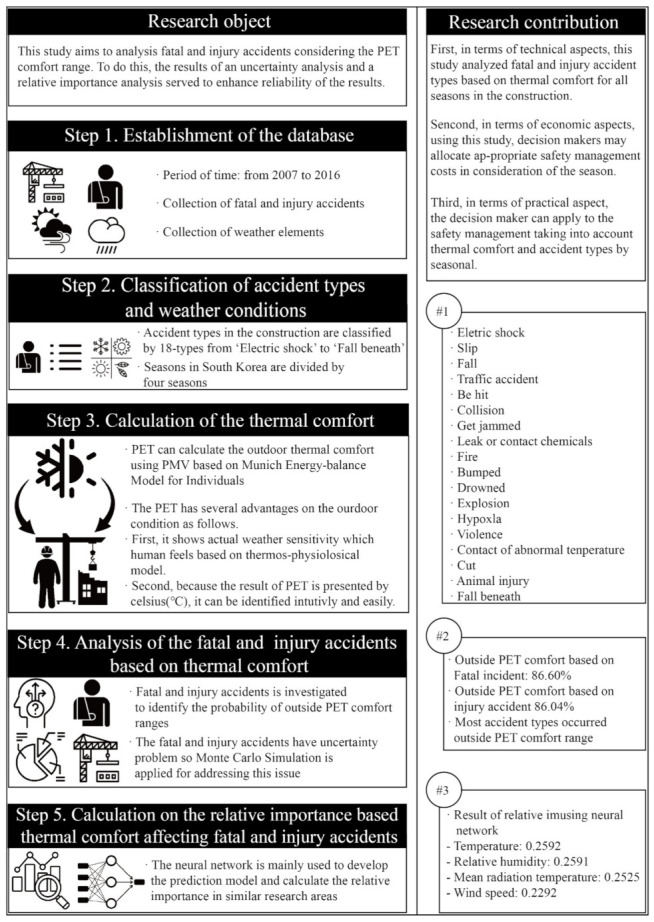
Research framework.

As shown in Figure 1, the research is conducted in five steps; (i) Establishment of the database, (ii) Classification of accident types and weather conditions, (iii) Calculation of thermal comfort, (iv) Analysis of injury and fatal accidents based on thermal comfort, and (v) Calculation of the relative importance of thermal comfort during injury and fatal accidents.

### 3.1. Establishment of the Database

Initially, information pertaining to 5318 fatal incidents and 207,802 injuries were collected from a national accident database in Korea as accident cases [49]. Climate information such as air temperature (T_a_), relative humidity (RH), and wind speed (*v*) were also collected from the Korea Meteorological Administration (KMA) to match the dates on which the accidents occurred [49].

Injury and fatal accidents are matched with the climate information considering location (from state to district) and time (from year to hour). Climate information from KMA is provided by region from city to district and time from year to hour. However, climate information by district remains the same. For example, referring to Table 2, when accidents occur in the same city, in different districts, and at the same time, the climate information remains the same. When accidents occur in the same city, the same district, and at different times, the climate information is applied differently. When accidents occur in different cities, different districts, and at the same time, climate information is applied differently.

In this study, the PET, a thermal comfort index, is utilized to analyze the weather impact on injury and fatal accidents. The input variables for the PET are T_a_, RH, and *v* and were obtained from the KMA, and the radiation temperature was calculated using RayMan Pro, a PET simulation tool [50].

### 3.2. Classification of Accident Types and Weather Conditions

Accident types and weather conditions can vary at construction sites during on-site work [31]. Thus, classifying accident types properly is important when analyzing the causes of accidents and determining how the weather conditions are related. In this study, accident types during construction work are classified into 18 types, e.g., ‘electric shocks’ and ‘fall beneath’, based on earlier work [49,51,52,53,54,55].

Korea has a monsoon climate and four seasons [56]. According to previous research, thermal comfort and related levels experienced by construction workers can differ depending on the season. Accident types can also differ depending on the level of thermal comfort [46,47,48]. In this regard, the seasons in Korea are divided into spring (March–May), summer (June–August), autumn (September–November) and winter (December–February) to analyze injury and fatal accidents and weather conditions [49].

### 3.3. Calculation of Thermal Comfort

A type of thermal comfort index known as the predicted mean vote (PMV) was presented by Fanger in 1970 as the ASHRAE standard 55 and the ISO 7730 standard [57,58,59,60,61,62,63,64,65,66,67]. PMV was developed to evaluate the thermal comfort of humans in a controlled IEQ using a HVAC system [57,58,59,60,64] (Equations (1)–(4)). Two types of factor, environmental factors and personal factors, should be considered to calculate thermal comfort. There are several environmental factors. Air temperature (T_a_) is the most important and is measured by the dry bulb temperature. Humidity is presented as the relative humidity (RH), which is the absolute humidity ratio to the maximum humidity in the air. Air velocity (v) is the wind velocity among indoor occupants. Because the indoor occupants are sensitive to the air velocity, this is an important factor affecting their thermal comfort. The mean radiant temperature (T_mrt_) is difficult to measure and therefore requires a globe thermometer to measure radiant heat directly from a warm object [60,68].

There are also several personal factors. The metabolic rate (M) is the value of the body surface area (W/m^2^). Clothing (Clo) is also one of the factors affecting heat dissipation which is directly related to the thermal performance of a person’s clothing [60,68].

(1) $$\mathrm{PMV}=\left(0.303e^{-0.036M}+0.028\right)\times \{\left(M-W\right)-3.05\times 10^{-3}\times \left[5733-6.99\left(M-W\right)-P_{a}\right]-0.0014M\left(34-t_{a}\right)\;-3.96\times 10^{-8}f_{\mathrm{cl}}\times {\left(t_{\mathrm{cl}}+273\right)}^{4}-f_{\mathrm{cl}}h_{c}\left(t_{\mathrm{cl}}-t_{a}\right)\}$$

(2) $$f_{\mathrm{cl}}=\left\{\begin{array}{c} 1.00+1.290\times I_{\mathrm{cl}}\;for\;I_{\mathrm{cl}}\;\leq 0.078{\;m}^{2}K/W\\ 1.05+0.645\times I_{\mathrm{cl}}\;for\;I_{\mathrm{cl}}>0.078{\;m}^{2}K/W \end{array}\right.$$

(3) $$t_{\mathrm{cl}}=35.7-0.028\left(M-W\right)-I_{\mathrm{cl}}\{3.96\times 10^{-8}f_{\mathrm{cl}}\times [{\left(t_{\mathrm{cl}}+273\right)}^{4}-\left(T_{\mathrm{mrt}}+273{)}^{4}\right]+f_{\mathrm{cl}}h_{c}\left(t_{\mathrm{cl}}-t_{a}\right)\}$$

(4) $$h_{c}=\left\{\begin{array}{c} 2.38{\left(t_{\mathrm{cl}}-t_{a}\right)}^{0.25}\;for\;2.38\times \left|t_{\mathrm{cl}}-{\left.t_{a}\right|}^{0.25}>12.1\sqrt{V_{\mathrm{ar}}}\right.\\ 12.1\sqrt{V_{\mathrm{ar}}}\;for\;2.38\times \left|t_{\mathrm{cl}}-{\left.t_{a}\right|}^{0.25}<12.1\right.\sqrt{V_{\mathrm{ar}}} \end{array}\right.$$

where PMV is the predicted mean vote, M is the metabolic rate, W is the effective mechanical power, P_a_ is the water vapor partial pressure, t_a_ is the air temperature, f_cl_ is the clothing surface area ratio to the body surface area, t_cl_ is the clothing surface temperature, I_cl_ is the thermal resistance of clothing, T_mrt_ is the mean radiant temperature, h_c_ is the convective heat transfer coefficient, and V_ar_ is the relative air velocity.

Unfortunately, PMV factors are focused on indoor thermal comfort; therefore, it is not appropriate to use PMV for outdoor thermal comfort based on previous research [57,58,59,60,61,62,63,64,65,66,67]. Hőppe presented the PET to address this issue. Referring to Table 3, the comfort range and variables of PMV and PET are presented [66]. Earlier researchers reviewed outdoor thermal comfort using the PET [69,70,71,72,73,74,75,76,77,78,79,80,81,82,83,84,85,86,87]. The PET has several advantages when used for outdoor conditions, as follows. First, it shows actual weather sensitivity as experienced by humans based on a thermo-physiological model. Second, the result of PET is presented in Celsius degrees (°C), which is intuitive and easy to identify. Third, the PET can be used to determine the differences in hot and cold climate. The authors determined that the PET is reasonable for evaluating the thermal comfort of construction workers, who typically stay outdoors.

The PET can be used to calculate the level of outdoor thermal comfort using PMV based on the Munich energy-balance model (Equation (5)) [76,77,78]. Generally, the PET requires a complex calculation process. Thus, previous research mainly used Rayman Pro, which as a simulation tool calculates the PET [85]. In this study, T_mrt_ and the PET are calculated using Rayman Pro. When calculating the PET using this software, the values of Clo and Met are correspondingly fixed at 0.9 Clo and 80 W as default values [84].

(5) M+WP+R+C+ED+ERe+ESw+S=0

where W_p_ is the physical work output, R is the net radiation of the body, C is the convective heat flow, E_D_ is the imperceptible perspiration, E_Re_ is the sum of the heat flow to heat and the humidity in inhaled air, E_Sw_ is the heat flow related to the evaporation of sweat, and S is the storage heat flow for heating or cooling the body mass.

### 3.4. Analysis of Injury and Fatal Accidents Based on Thermal Comfort

As discussed in Section 3.1, 5317 fatal incidents, 207,803 injuries, and weather data on the day an accident occurred for ten years were collected to analyze injury and fatal accidents while considering the thermal comfort factor in the construction industry. The numbers of injury and fatal accidents both contain some uncertainty. Accordingly, a Monte Carlo simulation (MCS) is applied to consider these uncertainties. MCS is a widely used method in risk management and provides probability distribution using random variables [88]. It is also used to calculate the probabilities of injury and fatal accidents which occur out of the range of thermal comfort in this case. MCS in this study is calculated using Equation (6) [89].

(6) $$E\left(X\right)\;≅\;\frac{1}{N}\sum_{n=1}^{N}x_{n},$$

where E(X) is the predicted random variable X, N is the number of random variables, and x_n_ is the random value.

### 3.5. Calculation on the Relative Importance of Thermal Comfort during Injury and Fatal Accidents

A neural network analysis, used in similar research areas, is conducted here to calculate the relative importance [90]. The neural network analysis uses a processing unit called a neuron. The neural network has three layers in this case: the input layer, the hidden layer, and the output layer [90]. The neural network is mainly used to develop the prediction model and calculate the relative importance.

Here, this type of network is used to calculate the variables of relative importance so as to identify the most influential environmental factor affecting injury and fatal accidents based on thermal comfort. Because the personal factors were fixed at 0.9 Clo and 80 W on RayMan Pro during the process of PET calculation, personal factors cannot be utilized to calculate the relative importance.

## 4. Result and Discussion

### 4.1. Analysis of Injury and Fatal Accidents Considering PET in South Korea

**Figure 2:**
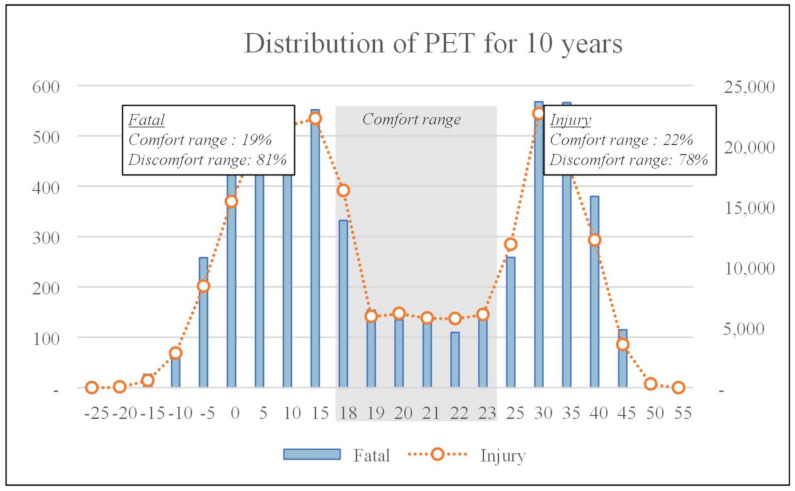
Distribution of the physiological equivalent temperature for 10 years.

**Figure 3:**
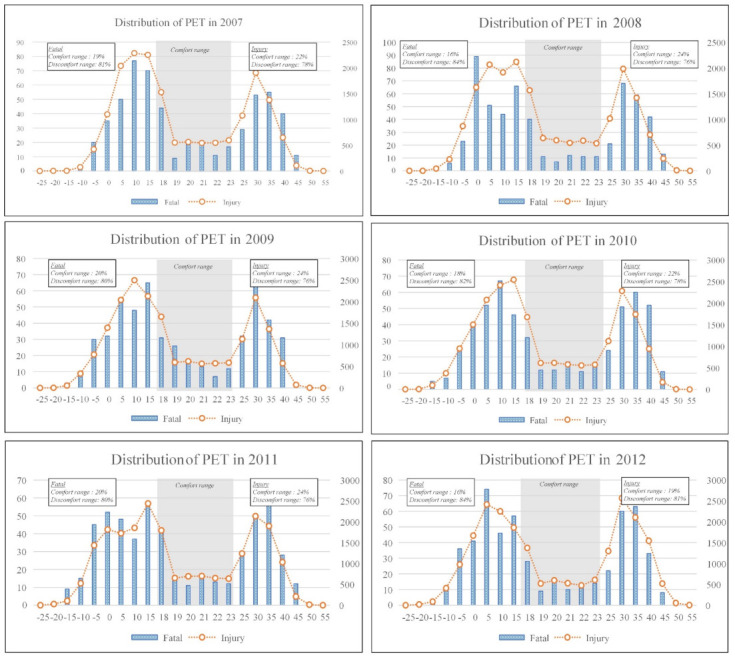

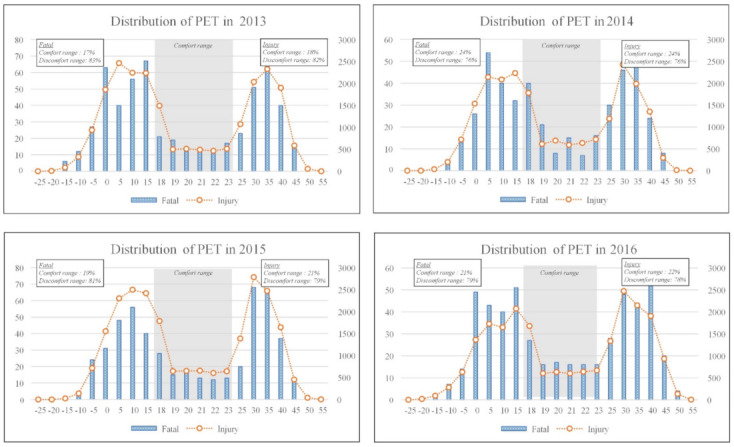
Distribution of physiological equivalent temperature from 2007 to 2016.

Before analyzing the PET of fatal and injury accident by considering accident type and month, the distribution of thermal comfort was analyzed for 10 years. As shown in Figure 2 and Figure 3, the distribution of PET is presented.

The discomfort range of fatal accident was from a minimum of 76% to a maximum of 84% yearly. Discomfort range for injury was from a minimum of 76% to a maximum of 82% yearly. For 10 years, the discomfort range of injury and fatal accidents occurred in the range of 81% and 78% in South Korea. Therefore, most construction workers have suffered fatal accident or injury accident within the discomfort ranges. So, thermal comfort should be maintained as the PET comfort range during construction work to eliminate and reduce injury and fatal accidents; however, this is very difficult.

According to Köppen climate classification, South Korea is classified as a monsoon climate which has four seasons from spring to winter. Weather conditions such as temperature experience a large gap between winter and summer from minimum −0.9 °C to maximum 25.4 °C (Refer to Table 4). PET range by month also witnesses a large gap from −12 °C to 21.6 °C. So, PET is almost distributed as a discomfort range by month, except for July and August.

Due to bad outdoor weather conditions, it is considered that more injury and fatal accidents have occurred in the construction industry than in other industries.

### 4.2. Analysis of Injury and Fatal Accidents Related to Outdoor Thermal Comfort

In this study, 18 accident types were analyzed to identify the relationship between injury and fatal accidents and thermal comfort. Before analyzing types of injury and fatal accident per month, the PET comfort distribution, and the probability distribution of injury and fatal accidents, ‘fall’ incidents were analyzed as an example to help readers understand the results. The ‘fall’ type represents the most common type of injury and fatal accident, at 2804 fatal accidents and 71,017 injury accidents. Therefore, the accident category ‘fall’ is representative among accident types.

**Figure 4:**
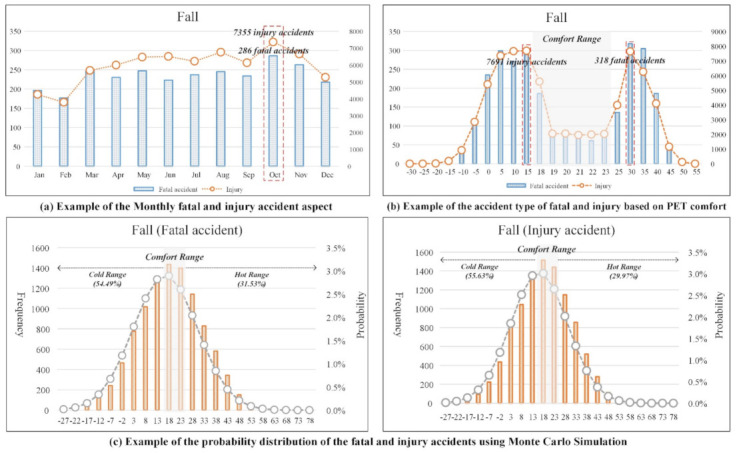
Examples of injury and fatal accidents in terms of thermal comfort.

First, as shown in Figure 4a, injury and fatal accidents due to falls were analyzed monthly. Injury and fatal accident occurred at nearly identical rates throughout the year. In winter (December to February), injury and fatal accidents occurred less than in other seasons. On the other hand, In October, the highest number of injury and fatal accidents occurred, with 286 fatal accidents and 7355 injury accidents.

Second, as shown in Figure 4b, ‘falls’ that led to injury and fatal accidents were analyzed based on PET comfort ranges. As mentioned above, the PET comfort range is defined from 18 °C to 23 °C. Injury and fatal accidents mainly occur outside the PET comfort range (excluding 18 °C to 23 °C). The most frequent type of fatal accident occurred from 25 °C to 30 °C, with 318 such accidents. The most frequent type of injury occurred from 10 °C to 15 °C, with 7691 injuries.

Third, as shown in Figure 4c, the probabilities of injury and fatal accidents that were ‘falls’ were presented using the probability distribution of PET ranges including both comfort and discomfort ranges. The probabilities of injury and fatal accidents that were ‘falls’ outside of the PET comfort range were 86.02% and 85.60%, respectively. It was identified that injury and fatal accidents of the ‘fall’ type show significantly high probabilities outside of the PET comfort range. Injury and fatal accidents also had high probabilities in the PET cold range, at 54.49% and 55.63%, respectively.

The analysis of injury and fatal accidents according to the 18 accident types in terms of the season, PET comfort range, and probability distribution are described in detail below.

### 4.3. Analysis of Monthly Injury and Fatal Accidents in Terms of Accident Type

**Figure 5:**
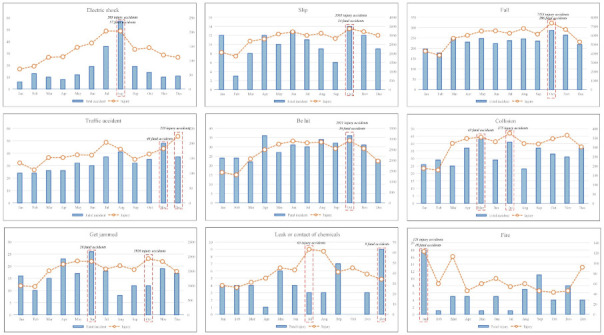

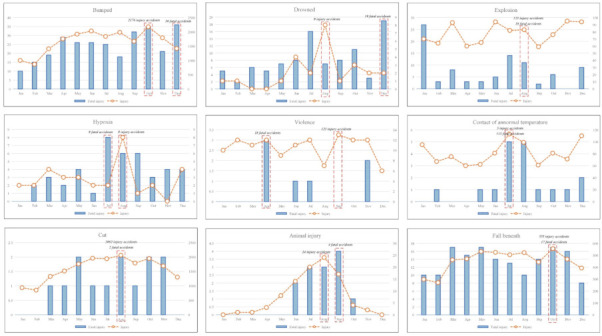
Graph of monthly injury and fatal accidents in terms of accident type.

As shown in Table 5 and Figure 5, injury and fatal accidents were analyzed by accident type in detail. The most common accident type, with a high frequency of injury and fatal accidents, is highlighted in boldface and gray. The accident types show significant differences relative to each other according to seasonal changes.

First, in the spring (March to May), three accident types (be hit, collision, and fall beneath) were most common among the 18 accident types. Specifically, there were 43 fatalities that were a ‘collision’ in May. This season (spring) has the fewest injuries among the four seasons.

Second, in the summer (June to August), four accident types (electric shock, hypoxia, contact of abnormal temperature, and cut) were most common. Particularly, there were 57 fatalities due to ‘electric shock’ in August. This season, summer, has the highest number of injuries, with eight accident types (electric shock, collision, leak or contact of chemicals, drowned, explosion, hypoxia, contact of abnormal temperature, cut, and animal injury) among the four seasons. Similarly to fatality cases, 203 injuries caused by an ‘electric shock’ in July and August represented the highest number for all injuries.

Third, in the autumn (September to November), six accident types were most common. Specifically, there were 286 fatalities caused by ‘fall’ in October. This season has the highest number of injuries, with eight accident types (slip, fall, be hit, get jammed, bumped, explosion, violence, and fall beneath) among the four seasons. Similarly to fatalities cases, 7355 injuries that were ‘fall’ in October represented the highest number of all injuries.

Fourth, in the winter (December to February), five accident types (traffic accident, fire, bumped, drowned, explosion) were most common. Specifically, there were 36 fatalities in the ‘bumped’ category in December. This season also has the highest number of injuries of two accident types (traffic accident and fire) among the four seasons. Additionally, 223 injuries caused by a traffic accident in December accounted for the highest number of all injuries.

Via these results, several reasons for the low number of accidents in summer and winter seasons can be considered, firstly, in terms of the number of construction workers. It may be regarded that the most injury and fatal accidents were caused by the larger number of construction workers. However, referring to Table 6, the number of construction workers is similar by month and the number of construction workers was not related to the number of injury and fatal accidents. For example, most construction workers worked in November; however, most injury and fatal accidents occurred in October. Second, in terms of working day, there are about 10 holidays per month in South Korea. There are the most working days in April and the fewest working days in May. So there is little relationship between working days and injury and fatal accidents. Third, in terms of PET by month, referring to Table 5, the PET was in the discomfort range in October; however, the PET was not worst in October. The lowest and highest PET were January and August, respectively. Therefore, the correlation between PET and accidents was weak.

### 4.4. Analysis of the PET Comfort Distribution in Terms of the Accident Type

**Figure 6:**
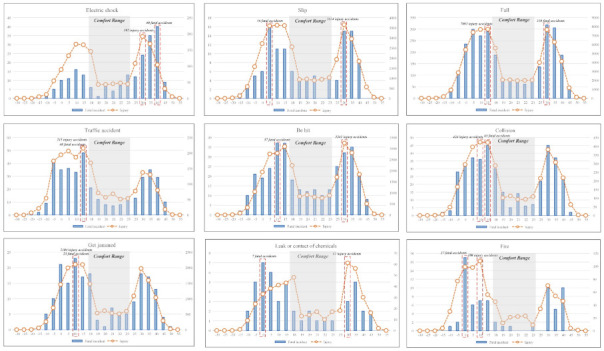

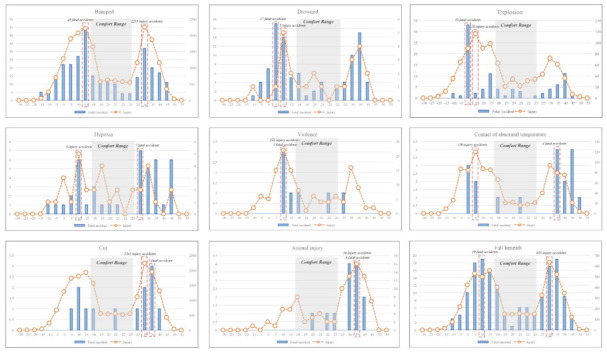
Graph of injury and fatal accidents types based on PET ranges.

As shown in Table 7 and Figure 6, injury and fatal accidents were analyzed based on comfort (18 °C~23 °C) and discomfort (excluding 18 °C~23 °C) PET range. The top 10% of the PET range for each accident type, with a high frequency of injury and fatal accidents, are highlighted as boldface and gray.

In Section 3.3, the PET comfort range was found to be 18 °C~23 °C. Table 6 shows that the most frequent injuries and fatal accidents occurred outside the PET comfort range for all accident types.

Most large fatal accident types occurred under the PET of 18 °C, including 12 types among the 18 accident types (Refer to Table 6). The most frequent accident type was ‘traffic accident’, which accounted for 48 fatalities from 10 °C to 15 °C. The most common injury types occurred under a PET of 18 °C, amounting to ten among the 18 accident types (Refer to Table 6). The most frequent accident type was ‘fall’, which accounted for 7691 injuries from 10 °C to 15 °C.

Most large fatal accident types occurred over a PET of 23 °C and there were six among 18 accident types (Refer to Table 6). The most frequent accident type was again ‘fall’, which had 318 fatalities from 25 °C to 30 °C. Most large injury accident types occurred over a PET of 23 °C and there were eight among the 18 accident types (Refer to Table 6). The most frequent accident type was ‘slip’, which numbered 3654 injuries from 25 °C to 30 °C.

### 4.5. Uncertainty Analysis of Accidents and Thermal Comfort

It was identified in the previous chapter that the most frequent injury and fatal accidents occurred outside the PET comfort range. Injury and fatal accidents fluctuate yearly in the construction industry. The probabilities of injury and fatal accidents under the PET comfort range are difficult to estimate using only historical data. Accordingly, MCS is applied to estimate the probability of accidents considering thermal comfort.

Referring to Table 8, the probabilities of injury and fatal accidents by accident type are presented using the probability distribution of the PET ranges, including both comfort and discomfort. The top accident types that are associated with high probabilities of injury and fatal accidents are highlighted in boldface and gray.

It was identified that injury and fatal accidents for every accident type show significantly high probabilities outside of the PET comfort range. Specifically, the probability of a fatality by ‘leak or contact chemicals’ outside of the PET comfort range was 88.91%, and the probability of injury by a ‘fire’ outside the PET comfort range was 88.95%. Both showed the highest probabilities among all accident types.

**Figure 7:**
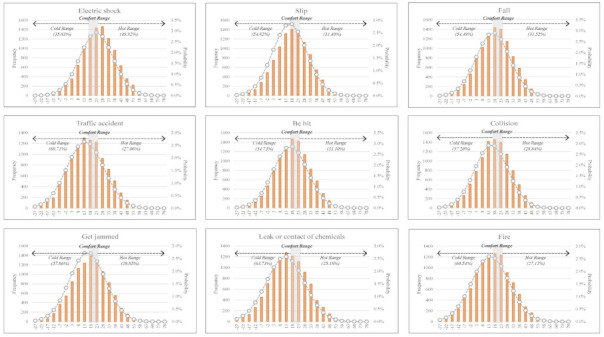

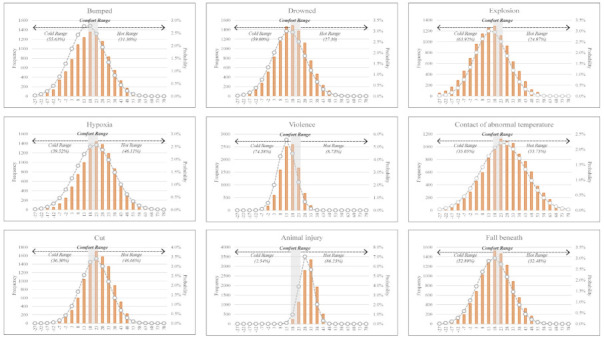
Analysis of the probability distribution of fatal accident using a Monte Carlo simulation.

**Figure 8:**
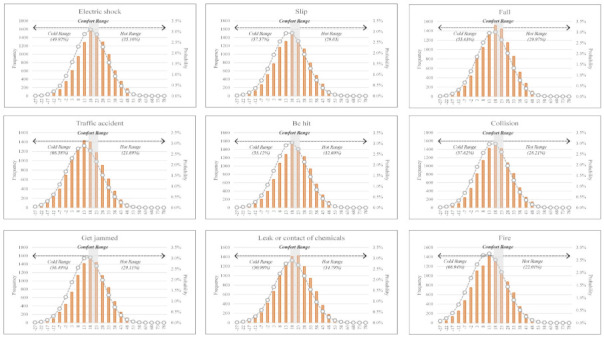

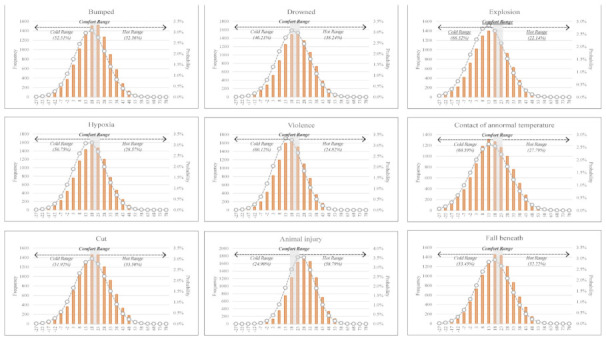
Analysis of the probability distribution of injuries using a Monte Carlo simulation.

In Figure 7 and Figure 8, the probability distributions of injury and fatal accidents are presented. In the PET cold range as part of the discomfort range, 13 accident types have a higher probability of fatality among the 18 accident types. The most common fatal accident types which have high probabilities in the PET cold range were in the ‘violence’ (74.58%), ‘explosion’ (63.92%), and ‘leak or contact chemicals’ (63.73%) categories. In addition, 17 accident types have a higher probability of injury among the 18 accident types. The most common injury accident types with high probabilities in the PET cold range were ‘fires’ (66.94%), ‘traffic accidents’ (66.58%), and ‘explosions’ (66.32%).

In terms of the PET hot range as part of the discomfort range, five accident types have higher probabilities of a fatality among the 18 accident types. The major fatal accident types which have a high probability in the PET hot range were ‘animal injury’ (86.23%), ‘contact of abnormal temperature’ (53.75%), and ‘electric shock’ (49.82%). Only the ‘animal injury’ category (58.79%) shows a higher probability in the PET hot range compared to the other ranges.

This result shows that the PET discomfort range is associated with a greater probability of accident than the PET comfort range and that the PET cold range means workers are more vulnerable than in the PET hot range in terms of the probabilities of injury and fatal accidents considering various accident types.

### 4.6. Relative Importance Analysis Based on Thermal Comfort of Injury and Fatal Accidents

In this study, a relative importance analysis was conducted to determine which environmental factor is more significant among T_a_, T_mrt_, v, and RH as input variables for the PET calculation.

**Figure 9:**
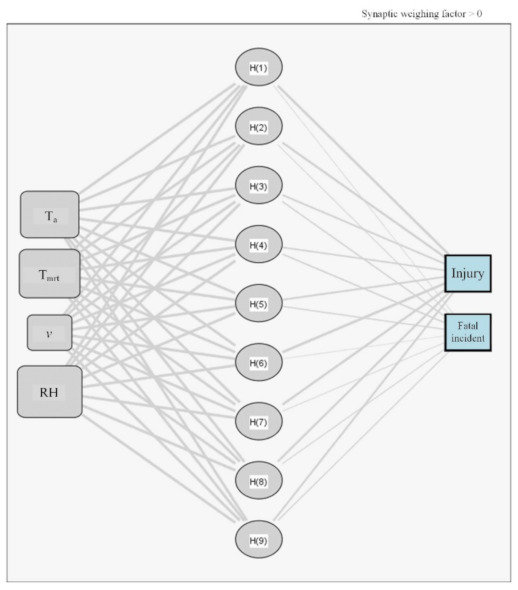
Graph of the neural network of weighting factors.

A neural network is used to calculate the environmental factors of the relative importance using SPSS 18. As shown in Figure 9, the training and test sets consisted of 70% and 30% of the total, respectively. Here, the training and test sets were typically split by 70% and 30% for analyzing the data set [91]. The input layer consists of T_a_, T_mrt_, v, and RH. There are nine hidden layers. The output layer consists of fatal incident and injury.

The variable R^2^ was calculated to validate the neural network model, with this result being 97.53%. Therefore, the proposed neural network model was validated. Referring to Table 9, the variables of the relative importance analysis are presented. The order of importance was T_a_ (0.2592), RH (0.2591), T_mrt_ (0.2525), and v (0.2292).

In this result, it was identified that T_a_ and RH are important environmental factors that influence injury and fatal accidents based on thermal comfort as compared to T_mrt_ and v.

### 4.7. Discussion

This study analyzed types of injury and fatal accidents affected by OEQ based on thermal comfort. Most of the earlier works in previous research focused on a specific weather condition, such as those in summer and winter seasons [4,5,6,7,8,9,10,11,12,13,14,15,16,17,18,19,20,21,22,23,24,25,26,27,28,29,30,31,32,33,34,35,36,37]. However, the results of this study showed that injury and fatal accidents in the spring and autumn seasons have greater probability than those of the summer and winter seasons. An expert interview was conducted to double-check this phenomenon, and related regulations pertaining to construction in South Korea were analyzed to interpret these results.

Based on the expert opinion and regulation review, the clients or the supervisor of the construction project have a duty and right to order a work stoppage when the weather is too hot or cold to proceed with common construction work (35 °C for summer and heavy snow accompanied by strong winds in winter (10 m/s) [92]. Thus, it can be assumed that injury and fatal accidents in summer and winter may occur less frequently than in other seasons, despite the fact that the probabilities of injury and fatal accidents in the PET discomfort range were significantly higher than the PET comfort range.

‘Fall’ was the most frequent accident type for both injury and fatal accidents, and this was proven throughout this study and in earlier research. Nadhim et al. (2016) insisted that weather and environmental conditions such as hot and cold temperatures and windy or rainy weather affect ‘fall’ accidents [93]. Gillen et al. (2002) identified weather is one of the important variables affecting ‘falls’ through a survey method [94].

Finally, a relative importance analysis was conducted to address the relative hierarchy among T_a_, RH, T_mrt_, and v as the input variables of the PET. In this study, the temperature is selected as the most important parameters. Previous research also mentioned the importance of the temperature in enhancing safety management [4,5,6,7,8,9,10,11,12,13,14,15,16,17,18,19,20,21,22,23]. High temperature can cause heat rash, heat exhaustion, and heatstroke in construction workers [4]. Low temperature in winter also can cause fatigue, lumbago, and cold finger sensation [6]. Rameezdeen and Elmualim (2017) insisted that heat stress causes heat rash, heat cramp, heat fainting, heat exhaustion and heatstroke in construction workers [4], Mohammed et al. (2019) noted how productivity losses, illnesses and injuries to workers may arise due to hot and humid weather conditions [13]. In addition, Yi and Chan (2013) found that hot and humid weather conditions caused worker fatigue and productivity losses, and they proposed an evaluation of heat stress to provide a proper balance between work and rest [16]. In this regard, it can be assumed that temperature can directly affect injury and fatal accidents in construction workers.

## 5. Conclusions

This study aimed to analyze injury and fatal accidents considering the PET comfort range. To achieve this, the results of an uncertainty analysis and a relative importance analysis were used to enhance the reliability of the results.

This study was conducted in five steps: (i) Establishment of the database, (ii) Classification of accident types and weather conditions, (iii) Calculation of the thermal comfort, (iv) Analysis of the injury and fatal accidents based on thermal comfort, and (v) Calculation on the relative importance of thermal comfort in affecting injury and fatal accidents.

The results of this study are as follows. First, the monthly analysis of injury and fatal accidents classified these incidents by accident type. There were more injury and fatal accidents in the autumn compared to the other seasons considering the 18 accident types. Six fatality types in the autumn were highest among the 18 accident types, and eight types of injury were highest among the 18 accident types. The most frequent injury and fatal accidents were ‘fall’ (286 fatal incidents and 7355 injuries). Second, all types of injury and fatal accidents occurred outside of the PET comfort range at significant levels, and these were defined as being in the PET cold and hot range. In the PET cold range (below PET 18 °C), the most frequent fatality types were ‘traffic accident’ (48 fatalities) from 10 °C to 15 °C. The most frequent injury was ‘fall’ (7691 injuries) from 10 °C to 15 °C. In the PET hot range (over PET 23 °C), the most frequent fatality type was ‘fall’ (318 fatal incidents) from 25 °C to 30 °C. The most frequent injury type was ‘slip’ (3654 injuries) from 25 °C to 30 °C. Third, the PET cold range was associated with a higher probability of injury and fatal accidents than the PET hot range for most types of fatality (13 of 18 accident types) and injuries (17 of 18 accident types). Finally, T_a_ (0.259) was the most influential factor in injury and fatal accidents among the four PET input variables according to the results of a relative importance analysis.

The contributions of this study are as follows. First, in terms of technical aspects, this study analyzed fatal and injury accident types based on thermal comfort for all seasons in the construction industry. Thus, this study can provide data for a database on safety management considering weather conditions. Second, in terms of economic aspects, using this study, decision makers may allocate appropriate safety management costs in consideration of the season. In terms of policy aspects, based on the results here, policymakers can legislate standards for seasonal safety management to reduce and eliminate injury and fatal accidents. Third, in terms of practical aspects, decision makers can apply these results to the safety management taking into account thermal comfort and accident types by seasonal. For example, the decision maker could plan safety management with respect to ‘Electric shock’, ‘Hypoxia’, and ‘Cut’ in the summer season, and ‘Traffic accident’ and ‘Fire’ in the winter season, because these occur more often during these seasons.

The limitations of this study are as follows. First, outdoor environmental quality has many factors, such as lighting, air quality, and noise. However, this study did not consider those factors. Second, there is a need to consider risk for construction workers according to their specific jobs, however; this study did not consider this kind of risk.

There are several directions for future research. First, injury and fatal accidents can be analyzed based not only on thermal comfort but also on other outdoor environmental qualities. Second, a prediction model can be developed to enhance safety management in the construction industry using machine learning.

## Figures and Tables

**Table 1:** Literature review and differences with this research.

Research Related to the Causes of Construction Disasters			
No.	Reference	Purpose	Difference
1	Berglund et al., 2019 [24]	The author analyzed fatalities and injuries according to daily, monthly, and workers’ ages in 2016 in Spain.	This study analyzes 18 accident types considering several weather conditions. These weather conditions affect construction workers’ thermal comfort.
2	Abukhashabah et al., 2020 [30]	The author investigated injuries and causes of incidents in the construction industry in Saudi Arabia, specifically Jeddah. A prevention method was presented to reduce injuries and incidents.	
3	Ahmed, 2019 [35]	The author sought to identify the causes of accidents at construction sites in Bangladesh and established the interests of workers, owners, consultants and contractors through questionnaire surveys.	
Research related to an analysis of the weather impact on injury and fatal accidents in construction			
4	Rameezdeen and Elmualim, 2017 [4]	The purpose of this study was to investigate heat waves and how they affect construction workers’ incidents from 2002 to 2013 in Australia.	This study investigates the link between thermal comfort and fatalities and accident incidents involving construction workers considering yearly weather conditions. Furthermore, using a neural network, relative importance is calculated and the effects on fatalities and injuries are determined.
5	Varghese et al., 2018 [9]	The author investigated heat-related illnesses such as heat stress and risk factors, associated diseases, and vulnerable groups in the construction industry.	
6	Acharya et al., 2018 [10]	The author presented evidence of a link between heat exposure and injuries. The result of this research provided policy proposals and directions for further research.	
Research related to measuring the thermal comfort of construction workers			
7	Yang, 2017 [46]	The author reviewed previous researches and categorized the methodologies related to thermal comfort assessments in the construction industry.	This study analyzes fatalities and injuries considering the PET. The probabilities of fatalities and injuries occurring outside the comfort range are also calculated.
8	Chan et al., 2012 [47]	The author developed a heat stress model based on the concept of the wet bulb globe temperature to measure the heat stress of workers.	
9	Yasmeen et al., 2020 [48]	The author analyzed the environmental and physiological factors affecting the ability and heat stress level in several building and work types in the construction industry.	

**Table 2:** Example of matching between accident data information and climate information considering city, district, and time.

City	District	Year	Month	Day	Hour	Temperature (°C)	T_mrt_ (°C)	Velocity (m/s)	Relative Humidity (%)	PET (°C)	Accident Type
Seoul	Gangbuk-gu	2009	1	2	9:00	−6.2	5.3	1.9	64	−9.5	Fall
Seoul	Jung-gu	2009	1	2	9:00	−6.2	5.3	1.9	64	−9.5	Traffic accident
Seoul	Gangseo-gu	2015	11	1	13:00	0.9	22.4	4.7	50	−2.5	Fall
Seoul	Gangseo-gu	2015	11	1	15:00	2.3	20.3	4.3	41	−1.3	Be hit
Cheongju	Sangdang-gu	2010	12	1	10:00	−1	17.2	4.5	77	−5	Fall
Incheon	Nam-gu	2010	12	1	10:00	−4.5	12.4	7.6	57	−10.5	Fall beneath

**Table 3:** PET variables and comfort range.

**Factor**		**Element**	**Range**
Environmental Factor		Temperature	−18.4 °C~39.3 °C
		T_mrt_	−32.1 °C~62.7 °C
		Relative humidity	0~100%
		Velocity	0 m/s~19.6 m/s
Personal Factor		Metabolic rate	80 W
		Clothing	0.9 Clo
**PET range**		**Thermal perception**	
Discomfort ranges (Cold ranges)	<4 °C	Very cold	
	4 °C~8 °C	Cold	
	8 °C~13 °C	Cool	
	13 °C~18 °C	Slightly cool	
Comfort range	18 °C~23 °C	Neutral	
Discomfort ranges (Hot ranges)	23 °C~29 °C	Slightly warm	
	29 °C~35 °C	Warn	
	35 °C~41 °C	Hot	
	41 °C	Very hot	

**Table 4:** Climate information and PET in South Korea for 10 years.

	January	February	March	April	May	June	July	August	September	October	November	December
Temperature (°C)	−0.9	1.6	6.3	12.2	17.8	21.6	25.1	25.4	20.9	15	8.2	1.3
Velocity (m/s)	2.3	2.4	2.5	2.5	2.2	2.0	2.0	1.9	1.7	1.9	2.1	2.3
Relative humidity (%)	60.8	60.0	59.2	61.2	63.7	72.9	81.0	79.4	76.5	71.3	67.5	63.3
PET (°C)	−12.0	−8.9	−2.8	4.9	12.2	16.9	21.2	21.6	16.5	9.3	0.5	−9.1

**Table 5:** Monthly analysis of injury and fatal accidents by accident type.

**Month**	**Electric Shock**		**Slip**		**Fall**		**Traffic Accident**		**Be Hit**		**Collision**		**Get Jammed**		**Leak or Contact of Chemicals**		**Fire**	
	**Fatal** **Incident**	**Injury**	**Fatal** **Incident**	**Injury**	**Fatal** **Incident**	**Injury**	**Fatal** **Incident**	**Injury**	**Fatal** **Incident**	**Injury**	**Fatal** **Incident**	**Injury**	**Fatal** **Incident**	**Injury**	**Fatal** **Incident**	**Injury**	**Fatal** **Incident**	**Injury**
1	6	70	12	2057	196	4250	24	134	24	1428	26	187	16	996	4	28	**18**	**123**
2	13	80	3	1847	177	3784	24	111	24	1310	29	178	10	967	4	26	1	60
3	10	111	8	2677	248	5674	26	152	22	2060	25	321	15	1507	4	31	5	113
4	8	113	12	2796	230	5990	26	152	**36**	2491	37	347	23	1731	1	35	5	46
5	12	146	10	3069	247	6464	32	161	27	2757	**43**	354	17	1845	6	45	1	60
6	19	161	13	3193	223	6499	30	160	31	2896	29	330	**26**	1831	4	43	5	70
7	36	**203**	11	2993	237	6218	37	203	30	2812	41	**375**	18	1570	3	**63**	1	54
8	**57**	**203**	9	3109	245	6751	41	180	34	2850	23	320	8	1685	3	61	7	60
9	19	139	6	2824	234	6126	32	146	32	2545	37	318	12	1549	**7**	41	11	46
10	14	145	**14**	**3365**	**286**	**7355**	35	164	**36**	**2921**	33	346	12	**1926**	0	45	4	43
11	10	119	12	3203	263	6641	**48**	182	31	2547	31	364	19	1826	3	39	8	46
12	11	111	9	2996	218	5266	37	**223**	23	1959	37	302	17	1488	9	34	4	92
Total	215	1601	119	34,129	**2804**	**71,078**	392	1968	350	28,576	391	3742	193	18,921	48	491	70	813
**Month**	**Bumped**		**Drowned**		**Explosion**		**Hypoxia**		**Violence**		**Contact of Abnormal Temperature**		**Cut**		**Animal Injury**		**Fall Beneath**	
	**Fatal Incident**	**Injury**	**Fatal Incident**	**Injury**	**Fatal Incident**	**Injury**	**Fatal Incident**	**Injury**	**Fatal Incident**	**Injury**	**Fatal Incident**	**Injury**	**Fatal Incident**	**Injury**	**Fatal Incident**	**Injury**	**Fatal Incident**	**Injury**
1	10	1000	5	1	**27**	70	0	2	0	10	0	95	0	935	0	0	10	297
2	15	864	2	1	3	64	2	2	0	12	1	67	0	852	0	1	10	290
3	19	1406	6	0	8	93	3	4	0	11	0	75	1	1327	0	1	**17**	461
4	29	1753	5	0	3	60	2	3	**3**	12	0	60	1	1515	0	3	15	470
5	26	1920	7	1	3	65	4	3	0	9	1	62	**2**	1758	0	8	**17**	531
6	26	2030	8	4	5	94	1	2	1	11	1	81	1	1957	2	14	14	524
7	25	1839	16	2	14	82	**8**	2	1	12	**5**	**113**	1	1939	3	20	13	504
8	18	1985	7	**8**	11	83	6	**8**	0	7	**5**	99	**2**	**2062**	3	**24**	10	522
9	32	1667	8	1	2	59	6	1	0	**13**	1	61	1	1791	**4**	17	14	442
10	35	**2176**	11	3	6	76	3	2	0	12	1	81	**2**	1946	1	4	**17**	**555**
11	21	1793	3	2	0	**95**	4	0	2	12	1	71	**2**	1688	0	2	16	467
12	**36**	1416	**19**	2	9	94	4	4	0	6	2	110	0	1301	0	0	8	392
Total	292	19,849	97	25	91	935	43	33	7	127	18	975	13	19,071	13	94	161	5435

Note: The most common accident type, with a high frequency of injury and fatal accidents, is highlighted in bold.

**Table 6:** Monthly analysis of the number of injury and fatal accidents, construction workers, and working and holiday from 2007 to 2016.

		January	February	March	April	May	June	July	August	September	October	November	December
Fatal accident	Mean	37.80	31.80	41.70	43.60	45.50	43.90	50.00	48.90	45.80	51.00	47.40	44.30
	Max	83.00	50.00	59.00	61.00	59.00	60.00	64.00	57.00	61.00	64.00	54.00	51.00
	Min	21.00	23.00	31.00	25.00	38.00	34.00	42.00	39.00	27.00	44.00	37.00	31.00
Injury	Mean	1168.30	1049.60	1602.40	1757.70	1925.80	1990.00	1900.00	2001.70	1778.60	2116.50	1909.70	1579.50
	Max	1414.00	1314.00	2076.00	1920.00	2185.00	2282.00	2169.00	2324.00	2130.00	2465.00	2099.00	2056.00
	Min	833.00	852.00	1258.00	1512.00	1601.00	1614.00	1495.00	1605.00	1257.00	1820.00	1700.00	406.00
Construction workers (Unit: 1000 workers)	Mean	1775	1749	1804	1854	1882	1894	1873	1852	1870	1881	1900	1871
	Max	1988	1964	1980	2023	2041	2056	2056	2031	2076	2090	2124	2074
	Min	1617	1575	1670	1735	1768	1776	1692	1681	1723	1686	1726	1701
Working day	Mean	20.70	18.70	21.40	21.60	20.40	20.50	22.30	21.20	19.50	20.60	21.30	21.60
	Max	22.00	21.00	22.00	22.00	22.00	22.00	23.00	22.00	22.00	21.00	22.00	23.00
	Min	19.00	17.00	20.00	20.00	19.00	19.00	21.00	20.00	17.00	20.00	20.00	20.00
Holiday *	Mean	10.30	9.50	9.60	8.40	10.60	9.50	8.70	9.80	10.50	10.40	8.70	9.40
	Max	12.00	11.00	11.00	10.00	12.00	11.00	10.00	11.00	13.00	11.00	10.00	11.00
	Min	9.00	8.00	9.00	8.00	9.00	8.00	8.00	9.00	8.00	10.00	8.00	8.00

Note: * Holiday includes weekends and national holidays.

**Table 7:** Distribution of injury and fatal accidents in terms of the PET range.

**PET (°C)**	**Electric** **Shock**		**Slip**		**Fall**		**Traffic** **Accident**		**Be Hit**		**Collision**		**Get** **Jammed**		**Leak or Contact** **Chemicals**		**Fire**	
	**Fatal** **Incident**	**Injury**	**Fatal** **Incident**	**Injury**	**Fatal** **Incident**	**Injury**	**Fatal** **Incident**	**Injury**	**Fatal** **Incident**	**Injury**	**Fatal** **Incident**	**Injury**	**Fatal** **Incident**	**Injury**	**Fatal** **Incident**	**Injury**	**Fatal** **Incident**	**Injury**
−30	0	0	0	0	0	0	0	0	0	0	0	0	0	0	0	0	0	0
−25	0	0	0	0	0	0	0	0	0	0	0	0	0	1	0	0	0	0
−20	0	0	0	22	1	20	1	7	0	10	0	1	0	4	0	0	0	0
−15	1	5	1	123	13	185	2	21	2	77	2	6	1	56	0	1	0	4
−10	1	12	3	583	33	920	9	54	10	332	3	48	5	257	2	9	1	30
−5	5	54	5	1575	115	2850	**42**	174	21	1048	28	165	10	706	5	24	2	77
0	10	89	6	2716	235	5408	35	194	19	1938	33	275	**21**	1455	**7**	33	**17**	**99**
5	11	132	**16**	3596	299	7350	36	**206**	24	2755	37	396	15	1993	**6**	38	6	98
10	16	168	11	**3625**	270	**7659**	33	186	**37**	2785	36	**424**	**23**	**2104**	3	41	7	**108**
15	13	166	11	3608	289	**7691**	**48**	**215**	**37**	**3089**	**45**	**422**	17	2098	5	43	7	56
18	6	145	6	2551	186	5594	21	168	18	2234	30	288	18	1475	2	48	2	45
19	1	43	4	932	86	2047	12	71	13	833	15	102	3	534	1	13	1	12
20	7	43	4	955	74	2056	8	57	12	903	5	113	1	608	2	14	1	21
21	4	45	5	892	68	1954	7	68	13	804	14	93	7	534	1	17	0	22
22	7	47	0	940	61	1988	8	51	10	761	6	93	5	511	1	10	0	0
23	13	44	5	1025	73	2023	13	53	13	876	7	109	5	594	1	17	1	9
25	12	108	4	1929	136	3993	13	77	25	1704	22	215	9	1086	0	18	0	34
30	24	**192**	**15**	**3654**	**318**	7645	29	137	32	**3241**	**45**	381	18	1971	3	**61**	**10**	70
35	**35**	**169**	**15**	2948	**305**	6262	35	130	35	2804	37	307	17	1591	5	**56**	5	54
40	**40**	104	8	1838	186	4102	29	80	21	1832	24	218	13	1035	2	30	**10**	46
45	9	30	0	565	53	1158	10	18	8	505	2	63	4	286	2	16	0	4
50	0	5	0	51	3	112	1	1	0	44	0	3	1	21	0	2	0	1
55	0	0	0	1	0	0	0	0	0	1	0	0	0	1	0	0	0	0
Mean (°C)	23.01	17.93	16.20	15.58	16.45	16.09	13.97	12.34	16.40	16.98	15.32	15.68	15.34	15.86	12.26	17.55	14.16	11.52
Median (°C)	25.30	18.8	18.40	16.50	17.50	17.00	13.55	13.40	18.40	18.10	15.90	16.30	15.90	16.60	11.00	18.70	12.90	9.20
25% (°C)	13.30	8.50	4.60	4.90	5.17	5.70	1.48	1.00	6.43	6.90	4.35	5.40	3.00	5.80	−0.38	7.20	−1.45	−0.50
75% (°C)	34.80	27.80	27.75	25.70	27.60	26.00	26.43	22.10	25.73	26.9	26.55	25.40	26.70	25.40	24.03	28.3	29.40	23.70
**PET (** **°C** **)**	**Bumped**		**Drowned**		**Explosion**		**Hypoxia**		**Violence**		**Contact of** **Abnormal** **Temperature**		**Cut**		**Animal** **Injury**		**Fall** **Beneath**	
	**Fatal** **Incident**	**Injury**	**Fatal** **Incident**	**Injury**	**Fatal** **Incident**	**Injury**	**Fatal** **Incident**	**Injury**	**Fatal** **Incident**	**Injury**	**Fatal** **Incident**	**Injury**	**Fatal** **Incident**	**Injury**	**Fatal** **Incident**	**Injury**	**Fatal** **Incident**	**Injury**
−30	0	0	0	0	0	0	0	0	0	0	0	0	0	0	0	0	0	0
−25	0	0	0	0	0	0	0	0	0	0	0	0	0	0	0	0	0	0
−20	1	2	0	0	0	3	0	0	0	0	0	0	0	6	0	0	0	3
−15	5	58	0	0	0	12	0	0	0	0	0	9	0	41	0	0	0	17
−10	4	257	1	1	2	35	1	1	0	2	0	26	0	233	0	1	3	75
−5	15	696	4	0	1	65	1	1	0	6	0	87	0	667	0	0	4	219
0	22	1279	7	0	**33**	90	1	3	0	5	3	84	0	1295	0	2	10	423
5	22	1896	**17**	1	2	**116**	2	1	0	15	2	**120**	1	1736	0	1	18	530
10	27	2072	14	**5**	4	90	**6**	**5**	**3**	**22**	0	87	**2**	1802	0	5	**19**	500
15	**45**	**2216**	5	2	11	98	1	2	**1**	15	0	85	1	1924	0	5	16	**559**
18	15	1646	6	1	4	63	3	2	**1**	8	1	66	1	1571	0	8	12	400
19	11	572	1	1	1	21	1	**4**	0	1	0	21	0	552	0	2	4	150
20	12	628	2	2	4	34	1	1	0	6	0	22	0	540	1	3	1	148
21	10	596	4	1	3	22	1	2	0	4	1	16	1	552	0	4	6	156
22	4	572	0	0	0	31	0	0	**1**	4	0	18	0	522	1	2	6	148
23	4	546	3	1	1	34	0	2	0	6	0	21	0	548	1	2	4	150
25	14	1155	4	1	2	43	**7**	2	**1**	4	0	40	1	1118	0	10	9	326
30	**32**	**2253**	10	3	4	72	5	**4**	0	**16**	0	**94**	**2**	**2241**	**4**	**13**	17	**635**
35	20	1867	**15**	**4**	6	61	**6**	1	0	9	**4**	79	**3**	**1951**	**4**	**16**	**19**	520
40	17	1158	4	2	**11**	36	1	0	0	2	2	75	1	1315	2	**13**	9	346
45	11	344	0	0	2	7	**6**	2	0	2	**4**	21	0	422	0	7	4	122
50	1	36	0	0	0	2	0	0	0	0	1	4	0	35	0	0	0	8
55	0	0	0	0	0	0	0	0	0	0	0	0	0	0	0	0	0	0
Mean (°C)	15.74	16.78	14.94	19.28	12.67	12.33	21.69	15.85	13.26	14.84	24.98	14.15	22.02	17.46	29.15	25.43	17.08	16.75
Median (°C)	15.95	17.70	17.20	20.00	12.30	12.70	24.10	18.10	13.80	14.60	33.10	14.40	24.50	18.50	28.80	25.20	17.70	18.00
25% (°C)	5.73	7.00	2.90	9.30	−3.10	1.20	9.75	6.10	6.35	6.45	4.85	1.80	10.70	7.30	16.60	18.60	17.55	6.00
75% (°C)	26.23	26.40	26.60	29.30	24.85	22.50	31.55	24.50	18.45	23.8	39.75	26.65	32.60	27.60	33.20	34.28	17.55	27.00

Note: The top 10% of the PET range for each accident type, with a high frequency of injury and fatal accidents, are highlighted in bold.

**Table 8:** Analysis of the probabilities of injury and fatal accidents in terms of the PET ranges.

**Fatal** **Incident**	**Electric Shock**	**Slip**	**Fall**	**Traffic Accident**	**Be Hit**	**Collision**	**Get Jammed**	**Leak or** **Contact Chemicals**	**Fire**	**Bumped**	**Drowned**	**Explosion**	**Hypoxia**	**Violence**	**Contact of** **Abnormal** **Temperature**	**Cut**	**Animal Injury**	**Fall** **Beneath**
Average (°C)	23.01	16.20	16.45	13.97	16.40	15.32	15.34	12.26	14.16	15.74	14.94	12.67	21.69	13.26	24.98	22.02	29.15	17.08
STD	13.60	13.92	13.71	15.05	13.85	13.52	14.49	15.49	15.35	14.16	13.18	15.60	13.99	7.18	17.96	11.67	5.57	13.26
Cold range (%)	35.83	**54.82**	**54.49**	**60.71**	**54.73**	**57.20**	**57.86**	**63.73**	**60.54**	**55.63**	**59.00**	**63.92**	39.52	**74.58**	35.05	36.30	2.54	**52.89**
Hot range (%)	**49.82**	31.40	31.52	27.06	31.10	28.84	29.85	25.18	27.12	31.30	27.30	24.97	**46.11**	8.73	**53.75**	**46.66**	**86.23**	32.48
Outside Comfort range (%)	85.65	86.22	86.01	87.77	85.83	86.04	87.71	88.91	87.66	86.93	86.30	88.89	85.63	83.31	88.80%	82.96	88.77	85.37
**Injury**	**Electric Shock**	**Slip**	**Fall**	**Traffic Accident**	**Be Hit**	**Collision**	**Get Jammed**	**Leak or** **Contact Chemicals**	**Fire**	**Bumped**	**Drowned**	**Explosion**	**Hypoxia**	**Violence**	**Contact of** **Abnormal Temperature**	**Cut**	**Animal Injury**	**Fall** **Beneath**
Average (°C)	23.01	16.20	16.45	13.97	16.40	15.32	15.34	12.26	14.16	15.74	14.94	12.67	21.69	13.26	24.98	22.02	29.15	17.08
STD	13.60	13.92	13.71	15.05	13.85	13.52	14.49	15.49	15.35	14.16	13.18	15.60	13.99	7.18	17.96	11.67	5.57	13.26
Cold range (%)	**49.92**	**57.57**	**55.63**	**66.58**	**53.12**	**57.62**	**56.49**	**50.99**	**66.94**	**52.51**	**46.21**	**66.32**	**56.75**	**60.12**	**60.59**	**51.92**	24.90	**53.45**
Hot range (%)	35.16	29.03	29.97	21.69	32.60	28.21	29.11	34.79	22.01	32.36	38.24	22.14	28.57	24.82	27.79	33.50	**58.79**	32.22
Outside Comfort range (%)	85.08	86.60	85.60	88.27	85.72	85.83	85.60	85.78	88.95	84.87	84.45	88.46	85.32	84.94	88.38	85.42	83.69	85.67

Note: The top accident types that are associated with high probabilities of injury and fatal accidents are highlighted in bold.

**Table 9:** Results of the variables of relative importance analysis.

Variables	Importance
T_a_	0.2592
T_mrt_	0.2525
v	0.2292
RH	0.2591

## Data Availability

The data presented in this study are available in the manuscript.
